# Uranium Speciation and Bioavailability in Aquatic Systems: An Overview

**DOI:** 10.1100/tsw.2002.130

**Published:** 2002-03-15

**Authors:** Scott J. Markich

**Affiliations:** Environment Division, Australian Nuclear Science and Technology Organisation, Private Mail Bag 1, Menai, NSW 2234, Australia

**Keywords:** analytical, bioavailability, freshwater, modeling, seawater, sediment, speciation, toxicity, uptake, uranium

## Abstract

The speciation of uranium (U) in relation to its bioavailability is reviewed for surface waters (fresh- and seawater) and their sediments. A summary of available analytical and modeling techniques for determining U speciation is also presented. U(VI) is the major form of U in oxic surface waters, while U(IV) is the major form in anoxic waters. The bioavailability of U (i.e., its ability to bind to or traverse the cell surface of an organism) is dependent on its speciation, or physicochemical form. U occurs in surface waters in a variety of physicochemical forms, including the free metal ion (U or UO_2_) and complexes with inorganic ligands (e.g., uranyl carbonate or uranyl phosphate), and humic substances (HS) (e.g., uranyl fulvate) in dissolved, colloidal, and/or particulate forms. Although the relationship between U speciation and bioavailability is complex, there is reasonable evidence to indicate that UO_2_ and UO_2_OH are the major forms of U(VI) available to organisms, rather than U in strong complexes (e.g., uranyl fulvate) or adsorbed to colloidal and/or particulate matter. U(VI) complexes with inorganic ligands (e.g., carbonate or phosphate) and HS apparently reduce the bioavailability of U by reducing the activity of UO_2_ and UO_2_OH. The majority of studies have used the results from thermodynamic speciation modeling to support these conclusions. Time-resolved laser-induced fluorescence spectroscopy is the only analytical technique able to directly determine specific U species, but is limited in use to freshwaters of low pH and ionic strength. Nearly all of the available information relating the speciation of U to its bioavailability has been derived using simple, chemically defined experimental freshwaters, rather than natural waters. No data are available for estuarine or seawater. Furthermore, there are no available data on the relationship between U speciation and bioavailability in sediments. An understanding of this relationship has been hindered due to the lack of direct quantitative U speciation techniques for particulate phases. More robust analytical techniques for determining the speciation of U in natural surface waters are needed before the relationship between U speciation and bioavailability can be clarified.

## INTRODUCTION

The bioavailability of uranium (U) (i.e., its ability to bind to or traverse the cell surface of an organism) is dependent on its speciation, or physicochemical form. U occurs in aquatic systems in a variety of physicochemical forms, including the free metal ion ($U^{4+}$ or $UO_2^{2+}$) and complexes with inorganic ligands (e.g., uranyl carbonate or uranyl phosphate), and humic substances (HS) (e.g., uranyl fulvate or humate) in dissolved, colloidal, and/or particulate forms[1]. Therefore, a knowledge of the distribution of U among its various physicochemical forms (i.e., speciation) is paramount to understanding the interaction of U with the cell surfaces of aquatic organisms (i.e., bioavailability). For the purposes of this review, a discussion of the speciation of U in relation to its bioavailability will be confined to surface waters (fresh- and seawater) and their sediments. Specialized reviews on the aqueous geochemistry of U should be consulted for additional information[1,2,3,4,5].

Surface waters and sediments contaminated with anthropogenic U, principally from the nuclear fuel cycle (e.g., mining and milling of U ore and reprocessing of waste) but also from the combustion of petrofuels (e.g., coal) and the manufacturing and application of phosphatic fertilizers (e.g., superphosphate), pose potential ecological risks[6,7]. Such risks are usually evaluated, at least in the first instance, by determining the total U concentration in water and/or sediments, and comparing these values with established guidelines or standards (where available) for protecting aquatic ecosystems. Despite U being radioactive, the chemical toxicity of U to aquatic organisms is of greatest environmental significance[8]. Analytical techniques used to measure total U (and its isotopic ratios) in natural surface waters ($<10\ \mu g\ l^{-1}$) and sediments have been extensively reviewed by Wolf[9]. These include atomic spectroscopy (e.g., graphite furnace atomic absorption spectrometry and fluorometry), mass spectroscopy (e.g., thermal ionization mass spectroscopy and inductively coupled plasma mass spectroscopy), and nuclear methods (e.g., $\alpha$-spectroscopy and neutron activation analysis).

U is one of the heaviest naturally occurring elements on Earth. It has 16 known isotopes, all of which are radioactive[10]. In nature, U consists of a mixture of three isotopes, $^{238}U$ (99.275%), $^{235}U$ (0.720%), and $^{234}U$ (0.005%). U is found in a wide range of rock types, with an average crustal concentration of 2.4 mg $kg^{-1}$[11]. Fine-grained sedimentary rocks typically have higher U concentrations than coarser-grained igneous rocks because of the higher content of clay and organic matter that readily binds U[12]. The geochemical cycle of U begins with the chemical weathering of rocks in the oxidized zone of the terrestrial near-surface environment, and continues with mobilization by ground and surface waters. During weathering, $^{238}U$ and $^{235}U$ are released to water in a constant isotopic ratio (137.88:1)[11]. $^{234}U$ is produced by the radioactive decay of $^{238}U$. However, the preferential mobilization of $^{234}U$ during weathering (due to $\alpha$-recoil fractionation) gives rise to a $^{234}U/^{238}U$ activity ratio greater than unity for river- (1.2 to 1.3) and seawater (1.14)[13].

U may occur in surface waters in three oxidation states: $U^{4+}$ (U[IV]), $UO_2^{+}$ (U[V]), and $UO_2^{2+}$ (U[VI] or uranyl ion). In anoxic waters (low redox potential), U occurs as $U^{4+}$ and/or $UO_2^{+}$. U(IV) has a strong tendency to precipitate (e.g., uraninite, $UO_2$[s]) and to remain immobile, whereas $UO_2^+$ forms soluble complexes[11]. In oxic waters, U occurs as $UO_2^{2+}$ and forms stable, readily soluble ionic and/or neutral complexes that are highly mobile and play the most important role in U transport during weathering[12]. The dominant U species are dependent on the pH-*E*h conditions and the concentration and availability of complexing ions[11]. The redox and complexation reactions of U are strongly influenced by hydrolysis, since hydrolytic reactions may limit the solubility or influence sorption to particles[14].

The average concentration of U (as $^{238}U$, the most abundant isotope) in riverwater is 0.3 µg $l^{-1}$[15,16] but typically ranges from 0.01 to 6.6 µg $l^{-1}$ depending on contact time with the U-bearing strata, the U content of the strata, the amount of evaporation, and the availability of complexing ions[11]. In estuaries, where river- and seawater mix, the concentration of dissolved U usually increases as a linear function of salinity (i.e., conservative behavior[16]), until it plateaus at around 3.2 µg $l^{-1}$ in seawater[13]. Although U usually behaves conservatively during estuarine mixing (e.g., Zaire, Gironde, Tama, and Medway estuaries), it may also behave nonconservatively, such as in the Ganges-Brahmaputra, and Amazon estuaries, where U is removed (sink) and added (source), respectively[16,17]. Riverwater is the only significant input of dissolved U (3 to $5 \times 10^7$ mol $year^{-1}$) to the oceans[15,16].

U concentrations in aquatic sediments typically range from 0.5 to 5 mg $kg^{-1}$, with an average of 3 mg $kg^{-1}$[6,18,19]. U concentrations may vary depending on the particle size, mineral composition (reflecting regional geology), and the physicochemistry of the water, particularly for freshwater environments. Substantial U enrichment (up to 100 times) has been reported for sediments sampled from anoxic environments (e.g., swamps or deep ocean basins)[20].

## SPECIATION METHODS

A plethora of reviews have been published on speciation methods for radionuclides and/or metals in surface waters and sediments[21,22,23,24,25]. The intention of this review is to summarize the methods that have been used to specifically determine the speciation of U. There is generally no accepted definition of *speciation*; various meanings have been attributed to the term by various workers. For the purposes of this review, speciation will be broadly defined as either (1) the process of identifying and quantifying the different defined species, forms, or phases present in a material; or (2) the description of the amounts and kinds of these species, forms, or phases present[21]. That is, the species, forms, or phases of U will be defined functionally, operationally, or as specific U species or oxidation states. Speciation methods may be classified into two categories:

1. *Analytical methods*, including physical separation (e.g., by species size and/or charge), electrochemistry, or spectroscopy; and
2. *Computational methods*, including thermodynamic (and kinetic) modeling.

### Analytical Methods

Analytical methods depend strongly on the nature of the medium (e.g., aqueous or particulate phases) to be analyzed and on the various species to be determined. Generally, no single method will provide unequivocal information on a metal's speciation. It is usually advantageous to combine two or more methods or use a speciation scheme[26]. However, there is no general consensus on whether one particular type of speciation scheme is better than another, since this will depend on the nature and character of the sample. It is important that the speciation of a metal is not altered following sample collection, storage, pretreatment, or by the analytical method itself (e.g., interference with equilibrium conditions). Analytical methods may be broadly grouped into (1) invasive techniques (i.e., samples that require pretreatment and/or separation), and (2) noninvasive techniques (i.e., samples that may be analyzed directly and do not require pretreatment and/or separation). The following U speciation techniques are generally restricted to those with demonstrated utility to natural surface waters (< 30 µg U $l^{-1}$) and solids (sediments).

## Water

One of the most important characteristics of U, like other metals, in surface waters is its distribution between particulate, colloidal, and dissolved forms (operationally defined as >450 nm, 1 to 450 nm, and <1 nm, respectively[22]). This is the most basic form of physical separation and discriminates U based on size, which is governed by the solubility of U and its affinity for the carrying particulate phase (driven by pH, *E*h, ligand concentration, etc.). Ultrafiltration, dialysis, gel filtration, and size exclusion chromatography have also been used to separate U species based on size (Table 1). Itoh et al.[27] extended the application of size exclusion chromatography by coupling it with inductively coupled plasma mass spectrometry (ICPMS) to quantify uranyl complexes with natural dissolved organic matter (DOM) in fresh surface water. Other physical speciation techniques, such as ion exchange chromatography and electrophoresis have been used to separate U species based on charge (Table 1). Rollin and Ecklund[28] used ion exchange chromatography coupled with ICPMS to separate $UO_2^{2+}$ and $U^{4+}$ at U concentrations typically found in natural surface waters. Pacheco and Havel[29] used capillary electrophoresis (CE) to identify uranyl complexation with humic acids. However, if CE were coupled with ICPMS, the increased sensitivity of the technique should be sufficient to quantify uranyl humate, or even uranyl fulvate, species, particularly in fresh surface waters.

Invasive electrochemical methods include voltammetry and potentiometry (Table 1). Cathodic stripping voltammetry (CSV) has been employed to measure labile (weakly complexed) uranyl species in estuarine and seawater at 0.5 to 3.5 µg $l^{-1}$[30]. van den Berg et al.[31] used adsorptive CSV (ACSV), an extension of CSV where a surface active chelating agent is added to a water sample to compete with natural complexing material (e.g., HS) for uranyl binding, to better differentiate labile and nonlabile U species. As a variant of this technique, Newton and van den Berg[32] employed adsorptive cathodic stripping chronopotentiometry (ACSC) in estuarine waters. Although the concentration of labile U species determined using ACSC is normally the same as that using ACSV, the former technique is generally less sensitive. The measurement of $UO_2^{2+}$ using ion selective potentiometry was found not to be feasible for natural surface waters[33], with detection limits of 5 to 20 mg $l^{-1}$.

The above-mentioned invasive speciation techniques are operational in nature and subject to artifacts, and the speciation results obtained are difficult to interpret with respect to bioavailable uranyl species. Time-resolved laser-induced fluorescence spectroscopy (TRLFS) is a unique, noninvasive, method (Table 1) for direct U speciation at concentrations typically measured in natural surface waters[34]. It has been used to identify and quantify $UO_2^{2+}$ and individual uranyl complexes with hydroxide, phosphate, sulfate, arsenate, and fulvate[34,35,36,37,38,39], based on calculated U speciation diagrams. While TRLFS is a promising technique for determining U speciation in natural waters, it is not suitable as a general method for all surface waters. One drawback of TRLFS is that fluorescence intensity decreases with increasing pH (from pH 5.5) and ionic strength at low U concentrations (<30 µg $l^{-1}$). The determination of higher uranyl carbonate species ($UO_2[CO_3]_2^{2-}$, $UO_2[CO_3]_3^{4-}$), for example, is difficult at pH 8.5[40,41]. Overall, TRLFS is not suitable at present for determining U speciation in freshwater at high pH or estuarine and seawater. No other direct speciation methods are currently available for measuring specific U species in natural surface waters. Techniques such as electrospray ionization mass spectrometry[42] or photothermal (displacement) spectroscopy[43] may prove useful for determining U speciation in natural waters after further development.

**TABLE 1**
**Analytical Methods Used to Determine the Speciation of Uranium (<30 µg $l^{-1}$) in Natural Surface Waters**

| Method | Uranium Species | Reference |
|---|---|---|
| Ultrafiltration | Dissolved and colloidal U in water | [110,111,112] |
| Dialysis | Dissolved and colloidal U in water | [113,114] |
| Gel filtration | $UO_2^{2+}$ and uranyl-humic complexes in water | [115] |
| Size exclusion chromatography (± inductively coupled plasma mass spectrometry) | Dissolved and colloidal U (U complexation with humic and fulvic acids) in water | [27] |
| Ion exchange chromatography (± inductively coupled plasma mass spectrometry) | Oxidation state (U(IV) or U(VI)) in water | [28,51] |
| Capillary electrophoresis | Uranyl complexation with humic and fulvic acids in water | [29,116] |
| Cathodic stripping voltammetry | Labile uranyl species in water | [31,117] |
| Cathodic stripping chronopotentiometry | Labile uranyl species in water | [32] |
| Time-resolved laser-induced fluorescence spectroscopy | $UO_2^{2+}$ and individual uranyl hydroxide, phosphate carbonate, sulfate, arsenate and fulvic acid complexes in water | [34,35,36,37,38,39] |
| Chemical (sequential) extraction | Operationally defined U fractions in solids | [86,87,88,89,90,91] |
| X-ray absorption near-edge structure spectroscopy | Oxidation state (U[IV] or U[VI]) in solids and water | [44,51] |
| Electron energy loss spectroscopy | Oxidation state (U[IV] or U[VI]) in solids | [52] |
| Optical luminescence spectroscopy | Coordination (near-neighbor) structure of $UO_2^{2+}$ in solids and water | [44] |
| X-ray absorption fine structure spectroscopy | Coordination (near-neighbor) structure of $UO_2^{2+}$ in solids and water | [54] |
| X-ray photoelectron spectroscopy | Oxidation state (U[IV] or U[VI]) and coordination (near-neighbor) structure of $UO_2^{2+}$ in solids | [53,55] |
| Photothermal (photoacoustic) spectroscopy | Coordination (near-neighbor) structure of $UO_2^{2+}$ in solids | [58] |

## *Sediment*

The mobility and bioavailability of U in solids (e.g., sediments) is often a function of the oxidation state (U[IV] or U[VI]), aqueous and solid phase chemistry, sorption to solid surfaces, and biogeochemical transformations[44]. Due to analytical difficulties, traditional methods for determining the speciation of U, and other metals, in sediments have relied primarily on chemical extraction techniques (Table 1), in which various phases within the matrix are operationally defined and interferences on chemical associations are generated[45]. Many different sequential extraction schemes have been developed (see reviews by Kersten and Förstner[46] and Kennedy et al.[47]). Chemical extraction techniques have been used, among other things, to indicate the fraction of total U in the sediment that is weak acid soluble (e.g., 0.1 to 1 M $HNO_3$ or HCl). For some metals of ecotoxicological relevance, such as Cd, Cu, Ni, Pb, and Zn, this metal fraction is positively correlated with the bioavailable metal fraction, under some environmental conditions[48,49]. This has not been demonstrated for U. Furthermore, in anoxic sediments, the toxicity of several metals to benthic organisms decreases as the concentration of acid-volatile sulfide increases[49].

Chemical extraction techniques are not ideal for determining the speciation of U (or most other metals) in solids (sediments), since it is not apparent what specific reactions take place during the chemical extraction of the chemically defined phases, nor what artifacts (e.g., metal redistribution) may be introduced during and/or following the extraction of a given phase. Furthermore, there has been no standardization of extraction techniques to directly compare the results of different studies. These limitations are minimized by using qualitative or quantitative *in situ* or direct speciation techniques. Some *in situ* spectroscopic techniques provide qualitative information that is selective for one oxidation state or mineral phase of U. *In situ* X-ray absorption spectroscopic techniques can provide detailed information about whether U is present as a particular oxide or sulfide phase. A select combination of these techniques may also be used to probe the speciation of U in sediments. For the determination of average U oxidation states (U[IV] or U[VI]) in sediments (solids), X-ray absorption near-edge structure spectroscopy (XANES) and electron energy loss spectroscopy (EELS) have been successfully applied (e.g., [44,50,51,52]) (Table 1). In conjunction with XANES, microprobe synchrotron X-ray fluorescence (micro-SXRF) and Auger electron spectroscopy have been used to investigate the homogeneity of U at sediment surfaces (e.g., [44,51,53]).

Optical luminescence spectroscopy (Table 1) is a powerful tool for the characterization of $UO_2^{2+}$ in nearly all matrices. Detailed speciation information on the coordination environment of $UO_2^{2+}$ can be gained from the luminescence spectra[44]. This technique provides many analytical advantages over other speciation probes. For example, it can be implemented in both spatially and temporally resolved modes that facilitate the detection and discrimination of U(VI) emission from that of other principally organic emitters found in sediments. The temporally resolved mode is particularly useful for matrices containing multiple uranyl species (e.g., complexes with inorganic and organic [HS] species). X-ray absorption fine structure spectroscopy (XAFSS) and X-ray photoelectron spectroscopy (XPS) have been used to provide information on the U oxidation state and the coordination environment of $UO_2^{2+}$ in solids (e.g., sediments) and at the solid-water interface (Table 1)[53,54,55]. Both in water and at the solid-water interface, the formation of multinuclear uranyl complexes may be detected as precursors to solid phase formation[56,57]. Fluorescent XAFSS is used for solids containing lower U concentrations, because of its higher sensitivity to U. Photothermal (photoacoustic) spectrometry (Table 1) has been used to effectively characterize the speciation of $UO_2^{2+}$ in solids, particularly amorphous phases[58].

## Computational Methods

Due to a lack of analytical methods to directly determine the speciation of U in natural surface waters, computational methods have predominantly been used. Much of the available information on the speciation of U in aquatic systems has been determined using thermodynamic speciation modeling. A speciation model is a mathematical statement (system of equations) of the methods and assumptions used to describe metal-ligand equilibria[59]. Two distinct, but thermodynamically related, methods are used by speciation models to calculate metal-ligand equilibria in aqueous systems: the equilibrium constant method and the Gibbs free-energy method[60]. Both methods are subject to the conditions of mass balance and chemical equilibrium. The mass balance condition requires that the calculated sum of the free and complexed species of each element be equal to the given total concentration. Chemical equilibrium requires that the most stable arrangement for a given system be found, as defined by the stability constants for all mass action expressions of the system, or through the use of Gibbs free-energy values for all components and derived species. In the equilibrium constant method, the mass action expressions are substituted into the mass balance equations, resulting in a set of nonlinear equations that must be solved simultaneously. The Gibbs free-energy method is simply a transformation of variables through the thermodynamic relation, which allows a different numerical approach[60].

A speciation code (e.g., MINTEQA2, PHREEQE, or MINEQL) is the practical realization of the solution to a speciation model (it executes calculations based on a model), typically written in a high-level computer language[60]. Most codes employ the equilibrium constant method, which uses measured or calculated stability constants for all mass action expressions of the system[61]. The model inputs include pH, redox potential, temperature, and total metal (e.g., U) and ligand (e.g., carbonate, sulfate) concentrations. As output, the percentage formation of relevant species, such as the free metal ion ($UO_2^{2+}$) and metal complexes (e.g., $UO_2CO_3$, $UO_2SO_4$), are calculated for specific physicochemical conditions[59].

Although a variety of speciation models are now widely available, all have significant limitations, and these are discussed in detail elsewhere[60,62,63]. One limitation is that most speciation models assume equilibrium conditions; i.e., the kinetics of precipitation, oxidation-reduction, and adsorption are generally ignored. This is not a valid assumption for U in some cases, because of kinetically unfavorable chemical processes, biological transformation, and physical transport[13]. There is a lack of kinetic-based speciation models available compared to thermodynamic (equilibrium) models. Although real systems are rarely at steady-state, this may be a more accurate approach than the common assumption of full equilibrium, which also lacks time resolution, but makes no allowance for reactions limited by chemical kinetics. STEADYQL[64] is an example of a speciation code that uses a steady-state box model to account for chemical kinetics, in addition to equilibrium and adsorption reactions. No data have been reported on the kinetic modeling of U in surface waters.

The modeling of U adsorption is not well developed in most speciation models, owing to a general lack of understanding of the phenomenon[65]. However, knowledge in this area is improving (see below). In addition, surface modifications may cause solids in natural systems to behave quite differently from pure phases[66]. Modeling of adsorption processes can be accomplished using stability constants, which are frequently estimated in the laboratory and later adjusted in field applications. Natural colloids are also very difficult to include in model calculations, and more work is required in this area.

The output from a speciation model is only as reliable as the input data. There is a clear requirement for a reliable and internally consistent database of stability constants to model chemical equilibria. Such a database exists for inorganic U species[61,67]. In natural waters, however, U binding by HS, in the form of fulvic and humic acids, is not accounted for by many speciation models, since these macromolecules are chemically ill defined and stability constants are poorly known. Markich and Brown[61] give an overview of the conceptual models that have been incorporated into some speciation codes to predict metal binding with HS, including U. HS may account for a significant portion of the U binding capacity of surface waters, and hence, markedly influence the speciation, transport, and bioavailability of U[68]. Therefore, U complexes with HS may be greatly underestimated, or not considered, in surface waters by many speciation models. As a result, such models may markedly overestimate the formation of inorganic U species.

The challenge when modeling the speciation of a metal, such as U, is to simulate the reactions between metal ions and dissolved, colloidal, and particulate chemical species in natural waters. Some speciation models have started to take into account the complex interactions of metals, including U, with HS, colloids, and suspended particles. The Windermere Humic Aqueous Model (WHAM), coupled with the Surface Chemistry Assemblage Model for Particles (SCAMP), is perhaps the most comprehensive integrated model to date[69,70]. WHAM models metal speciation in the dissolved phase, including metal interactions with HS, while SCAMP models metal interactions with natural particles. In the majority of literature examples, the integrated WHAM/SCAMP model has provided satisfactory agreement between observations and predictions of trace metal partitioning in natural waters[70]. However, to date, the model has not been evaluated with U, and this should be a priority for future research.

There is no general-purpose speciation model that can be used for all applications. While there are several limitations in using speciation models, the general consensus is that they can provide useful results if applied correctly and with an understanding of the differences between simulated and natural systems[65]. Only one study[71] has attempted to verify, in part, the speciation modeling results of dissolved U (in simulated freshwater) using an analytical technique (e.g., TRLFS).

## SPECIATION

### Freshwater

U is present as U(VI) in oxic fresh surface waters (pH 5 to 9). At environmentally relevant concentrations of dissolved U (<30 µg $l^{-1}$), the free uranyl ion ($UO_2^{2+}$) is calculated to be the predominant species at pH ≤ 5, but is insignificant at pH > 6 (Fig. 1). The formation of $UO_2OH^+$ is of secondary importance to $UO_2^{2+}$ at pH ≤ 5. An important complexing agent for U in oxic fresh surface waters is carbonate[72]. For fresh surface waters (0.3 µg U $l^{-1}$) with low hardness and alkalinity (<40 mg $CaCO_3$ $l^{-1}$) and very low natural DOM (<0.5 mg C $l^{-1}$), $UO_2CO_3$ is calculated to be the most dominant uranyl species from pH 5.5 to 6 (Fig. 1a). From pH 6 to 7.5, the mixed uranyl-hydroxide-carbonate species, $(UO_2)_2(OH)_3CO_3^-$, is calculated to be the most important uranyl species, while $UO_2(OH)_3^-$ is calculated to be the dominant uranyl species at pH > 7.5 (Fig. 1a). A 100-fold increase in U concentration (i.e., 30 µg $l^{-1}$), which encompasses most natural waters impacted by anthropogenic U sources (e.g., mining), results in a shift in the percentage distribution of U (Fig. 1b). The major difference is that $(UO_2)_2(OH)_3CO_3^-$ is the most dominant uranyl species from pH 5 to 8.5 (Fig. 1b).

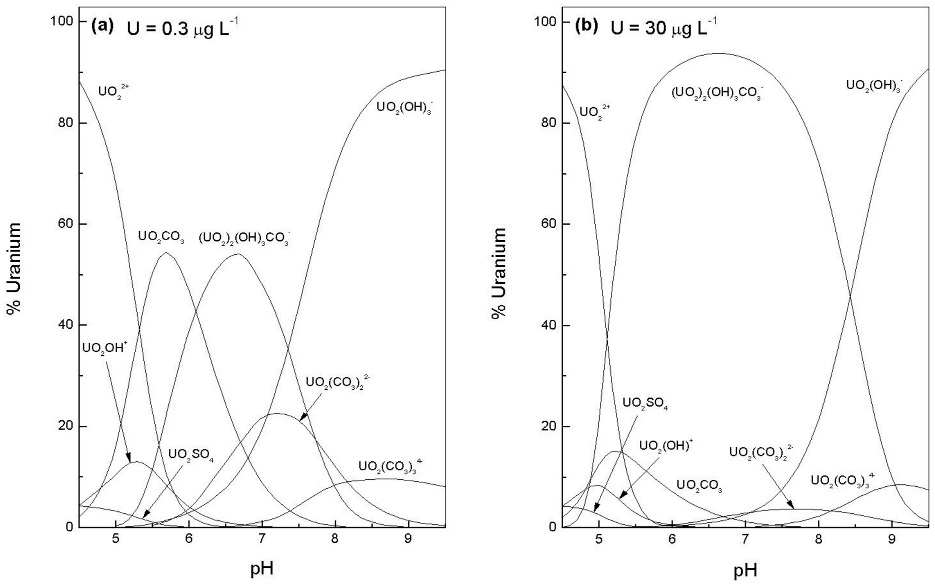

**FIGURE 1.** Predicted speciation (% distribution) of U as a function of pH (4.5 to 9.5) for a model freshwater at (a) 0.3 µg U $l^{-1}$ and (b) 30 µg U $l^{-1}$ without humic substances. U species <2% are excluded for clarity. U speciation was calculated using HARPHRQ and based on the ionic composition of the Hawkesbury-Nepean River, Sydney, Australia[118] (carbonate = 40 mg $l^{-1}$; sulfate = 9.4 mg $l^{-1}$; chloride = 5.1 mg $l^{-1}$; ionic strength = 0.002 M). Stability constants for U at 25°C were taken from Markich and Brown[61].

In natural fresh surface waters, with U concentrations <30 µg $l^{-1}$, the formation of polymeric uranyl hydroxide species, such as $(UO_2)_2(OH)_2^{2+}$, $(UO_2)_3(OH)_5^{+}$, $(UO_2)_4(OH)_7^{+}$, and $(UO_2)_3(OH)_7^{-}$, is predicted to be insignificant (<1% of total U). These species start to become more important at U concentrations >200 µg $l^{-1}$. Uranyl complexes with chloride, nitrate, silicate, sulfate, and fluoride are relatively weak in comparison to carbonate and phosphate[67]. Uranyl phosphate complexes, however, are usually of minor importance in most natural fresh surface waters, because phosphate concentrations are typically very low (<10 µg $l^{-1}$).

In addition to carbonate, natural DOM, in the form of HS (fulvic and humic acids), is a very effective complexing agent of U in fresh surface waters[14,73]. HS may be soluble or insoluble, depending on molecular weight, state of aggregation, degree of protonation, the extent of U binding, and the ionic strength of the water. They may act as a sink for U, if the uranyl-HS complex is insoluble, or as a mobile phase, if the uranyl-HS complex is soluble[74]. In organic-rich fresh surface waters (pH 4.5 to 8) with low hardness and alkalinity (<40 mg $CaCO_3$ $l^{-1}$), uranyl complexes with HS are the dominant species of dissolved U (Fig. 2). Complexation is pH dependent; it typically increases up to about pH 6 because of increasing ionization of the carboxylate (COOH) functional groups of HS[14], but then decreases markedly because of a higher binding affinity with carbonate and hydroxide (e.g., increased formation of $UO_2[OH]_3^{-}$, $UO_2[CO_3]_3^{4-}$, $UO_2[CO_3]_2^{2-}$, and/or $[UO_2]_2[OH]_3CO_3^{-}$; Fig. 2). The formation of U complexes with HS generally decreases, with increasing U concentration, once the binding saturation of the HS by U is reached[75]. Uranyl-carbonate and uranyl-hydroxide-carbonate species become more important than uranyl complexes with HS as the hardness, alkalinity, and pH of the water increase (usually > pH 7)[73]. This is exemplified for pH in Fig. 2.

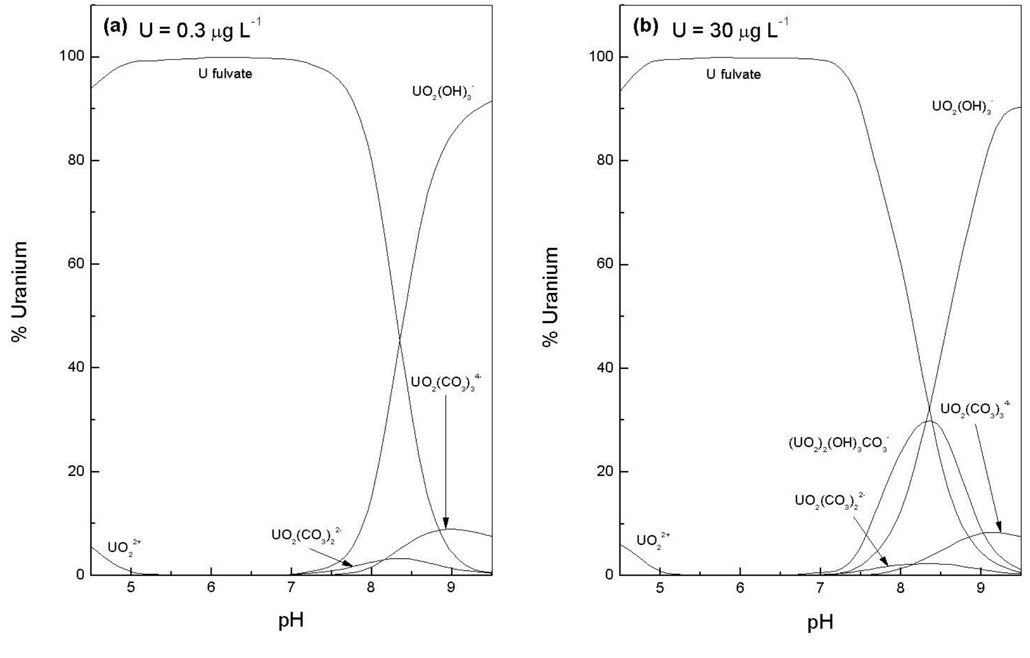

**FIGURE 2.** Predicted speciation (% distribution) of U as a function of pH (4.5 to 9.5) for a model freshwater at (a) 0.3 µg U $l^{-1}$ and (b) 30 µg U $l^{-1}$ with HS (4.2 mg $l^{-1}$ fulvic acid; 4.5 mg $l^{-1}$ dissolved organic carbon). U species <2% are excluded for clarity. U speciation was calculated using HARPHRQ and based on the ionic composition of the Hawkesbury-Nepean River, Sydney, Australia[118] (carbonate = 40 mg $l^{-1}$; sulfate = 9.4 mg $l^{-1}$; chloride = 5.1 mg $l^{-1}$; ionic strength = 0.002 M). Stability constants for U at 25°C were taken from Markich and Brown[61].

Sorption plays a dominant role in determining the fate of U in freshwater systems. Sorption to clay minerals (e.g., smectite or montmorillonite) below pH 5, and to Fe and Al (oxy)hydroxides, silica, and biotic surfaces, at higher pH, reduces the mobility of U in oxic freshwaters[76,77,78]. Sorption of U to insoluble organic matter, or organic matter attached to particles (e.g., hydrous iron oxides), also reduces the mobility of U[79]. It is generally established that sorption of U to particles increases with increasing pH until a threshold point is reached[80], which varies as a function of the concentration of U, adsorbent, competing ions (e.g., carbonate), chelating agents, and ionic strength[81]. In fresh surface waters at pH 6 to 8, the solubilities of U(VI) minerals are near their minimum[11] and the sorption of uranyl by HS near its maximum[68].

In anoxic fresh surface waters, U(IV) is the predominant oxidation state of U. It hydrolyzes at very low pH (~1) and has a very low solubility (and hence mobility) in the circumneutral pH range encountered in most fresh surface waters[1]. However, at pH > 8, there is some evidence that U(IV) forms soluble complexes with carbonate (as $U[CO_3]_3^{2-}$ and $U[CO_3]_5^{6-}$) and natural DOM, if present at sufficiently high concentrations[1]. U(IV) may be oxidized to U(VI); $U^{4+} + 2H_2O \Leftrightarrow UO_2^{2+} + 4H^+ + 2e^-$; $E^o = -268$ mV[67].

## Seawater

In oxic seawater (pH ~8) U is unreactive and exists predominantly (80 to 90%) as a stable, soluble, uranyl tricarbonate complex, $UO_2(CO_3)_3^{4-}$, that is inefficiently scavenged by particulate matter[82]. In the photic zone, where a relatively high concentration of hydrogen peroxide ($H_2O_2$) is found, U shows a strong binding affinity for the peroxide ligand, forming minor proportions (10 to 20%) of the mixed uranyl dicarbonate hydrogen peroxide complex, $UO_2(CO_3)_2(HO_2)^{3-}$[83]. The actual proportions of $UO_2(CO_3)_2(HO_2)^{3-}$ are governed by the concentration of $H_2O_2$ in seawater. The photic zone is also relatively rich in natural DOM, where uranyl complexes with HS form a small, but significant, component (<20%) of the dissolved U concentration[84]. This component is strongly dependent on the concentration of natural DOM, which is typically 0.5 to 1.2 mg $l^{-1}$. Since the U concentration of seawater is typically low (around 3 μg $l^{-1}$), polymeric uranyl hydroxides are not significant, and monomeric uranyl hydroxides are less important than uranyl carbonate complexes[85]. In anoxic waters, U(IV) forms insoluble polymeric mixed hydroxides and carbonates, which deposit on the seabed (i.e., U[IV] is extremely reactive with respect to adsorption and scavenging by particulate matter). Choppin and Wong[4] give a more detailed review of the behavior of U and other actinides in seawater.

## Sediments

A number of studies[86,87,88,89,90,91] have employed sequential extraction to indirectly determine the speciation of U in sediments or suspended particulate matter. Howe et al.[87] used a three-stage sequential extraction protocol[92] to partition U from Whitehaven Harbour (U.K.) sediment into exchangeable (water and acid soluble), oxidizable (bound to Fe and Mn oxyhydroxides), reducible (bound to organic matter and sulfides), and residual (insoluble) fractions. Although the distribution of U in the sediment samples was somewhat variable, it was predominantly (40 to 80%) associated with the oxidizable fraction. Desideri et al.[89] used a five-stage sequential extraction protocol[93] to partition U from several marine sediments into water soluble, acid soluble, carbonate, reducible, and residual sediment fractions. In contrast to the results of Howe et al.[87], U was associated primarily with the carbonate and residual sediment fractions.

Kaplan and Serkiz[90], using a slightly different five-stage sequential extraction procedure[94], found that U was associated predominantly (90%) with the residual phase of an uncontaminated sediment (i.e., U was strongly bound). Conversely, U in contaminated sediments was associated predominantly (90%) with the exchangeable and reducible (organic matter) phases (i.e., U was weakly bound). Hirose and Sugimura[86] used a four-stage sequential extraction protocol to partition U in suspended particulate matter from the North Pacific Ocean. The majority (~60%) of U was associated with the organic binding sites of particles. In the only study of freshwater sediment, Leleyter and Probst[88] used a seven-step sequential extraction procedure to partition U in sediment from the Garonne River (France). The authors found that U was associated mainly with the iron oxide (54%) and carbonate (28%) sediment phases, with only 4% being regarded as exchangeable.

Overall, chemical extraction methods provide an ambiguous approach to evaluating the speciation of U in sediment. The variability in the partitioning of U in the sediments/particles in the above-mentioned studies may stem from (a) using nonstandardized extraction methods, (b) the source of U (anthropogenic vs. natural-occurring U), and/or (c) differing sediment composition (e.g., variables percentages of Fe/Mn oxyhydroxide or organic matter). Ideally, quantitative *in situ* X-ray spectroscopic techniques could provide a superior and more objective measure of the speciation of U bound to/in solids. In practice though, a three-orders-of-magnitude improvement in current technology is needed. Moreover, X-ray spectroscopy gives only an average result, so where there are mixtures of U species, only the concentration-weighted average coordination environment can be measured—the result cannot be deconvoluted into separate components.

## BIOAVAILABILITY

### General

U has no known essential role in the normal biochemical reactions that occur in organisms. However, there is evidence that U is taken up at the cell surface in mistake for Ca, an essential metal[71,95]. Bioassays are typically used to ascertain U-organism interactions. These can be coupled with the measured and/or predicted speciation of U to determine bioavailable U species.

There is little information relating the speciation of U to its bioavailability in aquatic systems. All available information has been derived using simple, chemically defined experimental freshwaters rather than natural waters. No data are available for estuarine or seawater. Furthermore, there are no available data on the relationship between U speciation and bioavailability in sediments. These two gaps clearly require further work. The bioavailability of U in freshwater is influenced by a variety of physicochemical variables, including pH, HS, water hardness, and alkalinity[96,97,98,99,100].

### Effects of pH and Humic Substances

Few studies have reported on the effect of pH and/or HS on U uptake by, or toxicity to, freshwater organisms. Markich et al.[98] found that the valve movement responses (measured in terms of the duration of valve opening, or DVO) of the freshwater mussel, *Velesunio angasi*, exposed to U in experimental (synthetic) Magela Creek water (Australia) were highly dependent ($p \leq 0.001$) on the pH and/or the concentration of DOM (expressed in the form of a model fulvic acid [FA], comprising aspartate, citrate, malonate, salicylate, and tricarballyate in defined ratios). For a given model FA concentration, the toxicity of U to *V. angasi* decreased exponentially as the pH increased from 5 to 6. Similarly, for a given pH, the toxicity of U to *V. angasi* decreased exponentially as the model FA concentration increased from 0 to 7.9 mg $l^{-1}$.

In the absence of model FA, the toxicity of U to *V. angasi* decreased fivefold with an increase in pH from 5 to 6 (Fig. 3). For example, the 48 h $EC_{50}$ (i.e., the concentration of U that reduced the DVO by 50%) increased from 103 μg $l^{-1}$ at pH 5 to 556 μg $l^{-1}$ at pH 6. Additionally,

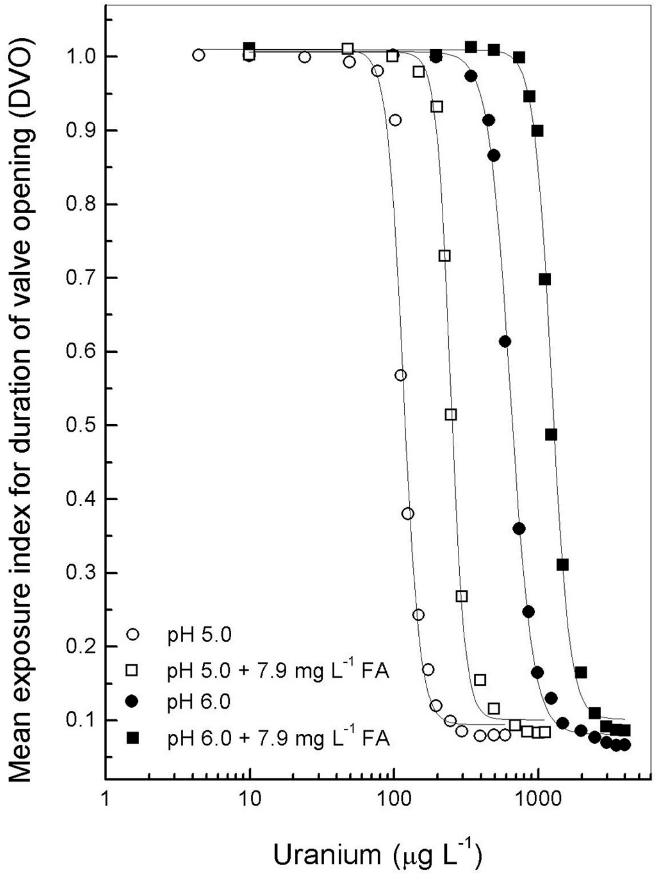

**FIGURE 3.** Concentration-response relationships of the duration of valve opening (DVO) for *V. angasi* exposed to U in synthetic Magela Creek water at pH 5 and 6 with and without model FA (7.9 mg $l^{-1}$). Each plotted point represents the mean response of six bivalves. Error bars are excluded for clarity.

in the presence of the maximum concentration of model FA (7.9 mg $l^{-1}$), the toxicity of U to *V. angasi* at pH 5 and 6 was reduced twofold (Fig. 3). For example, the 48 h $EC_{50}$ increased from 556 µg $l^{-1}$ at pH 6 without model FA to 1080 µg $l^{-1}$ at pH 6 with 7.9 mg $l^{-1}$ FA. The toxicity of U to *V. angasi* at a given total U concentration was greatest at pH 5 without model FA, and least at pH 6 with 7.9 mg $l^{-1}$ of model FA.

The speciation of U at pH 5 without model FA and pH 6 with 7.91 mg $l^{-1}$ FA was calculated using the speciation code HARPHRQ[101]. In summary, at pH 5 without model FA, $UO_2^{2+}$ (43 to 58%) and $UO_2OH^+$ (26 to 36%) were the dominant uranyl species predicted to form (Fig. 4a). The relative proportions of these two species decreased with increasing total U concentration; such decreases were offset by increases in the relative proportions (0 to 12%) of polymeric uranyl species (i.e., $[UO_2]_2[OH]_2^{2+}$ and $[UO_2]_3[OH]_5^{+}$). In contrast, the proportions of $UO_2^{2+}$ (2 to 4%) and $UO_2OH^+$ (8 to 12%) formed a minor contribution to the total U concentration at pH 6 with 7.9 mg $l^{-1}$ FA (Fig. 4d). As expected, the predicted speciation of U was altered by the addition of model FA, with the formation of three organic uranyl species ($UO_2[OH]Cit^{2-}$, $UO_2Mal$, and $UO_2[OH]Mal^-$, where Cit is citrate and Mal is malonate). The predicted concentrations of $UO_2^{2+}$ and $UO_2OH^+$ at pH 5 without model FA, were in close agreement (2 to 5% difference) with those measured using TRLFS. Using multiple linear regression analysis, combined with speciation modeling, Markich et al.[98] provided evidence to show that, under the prescribed experimental

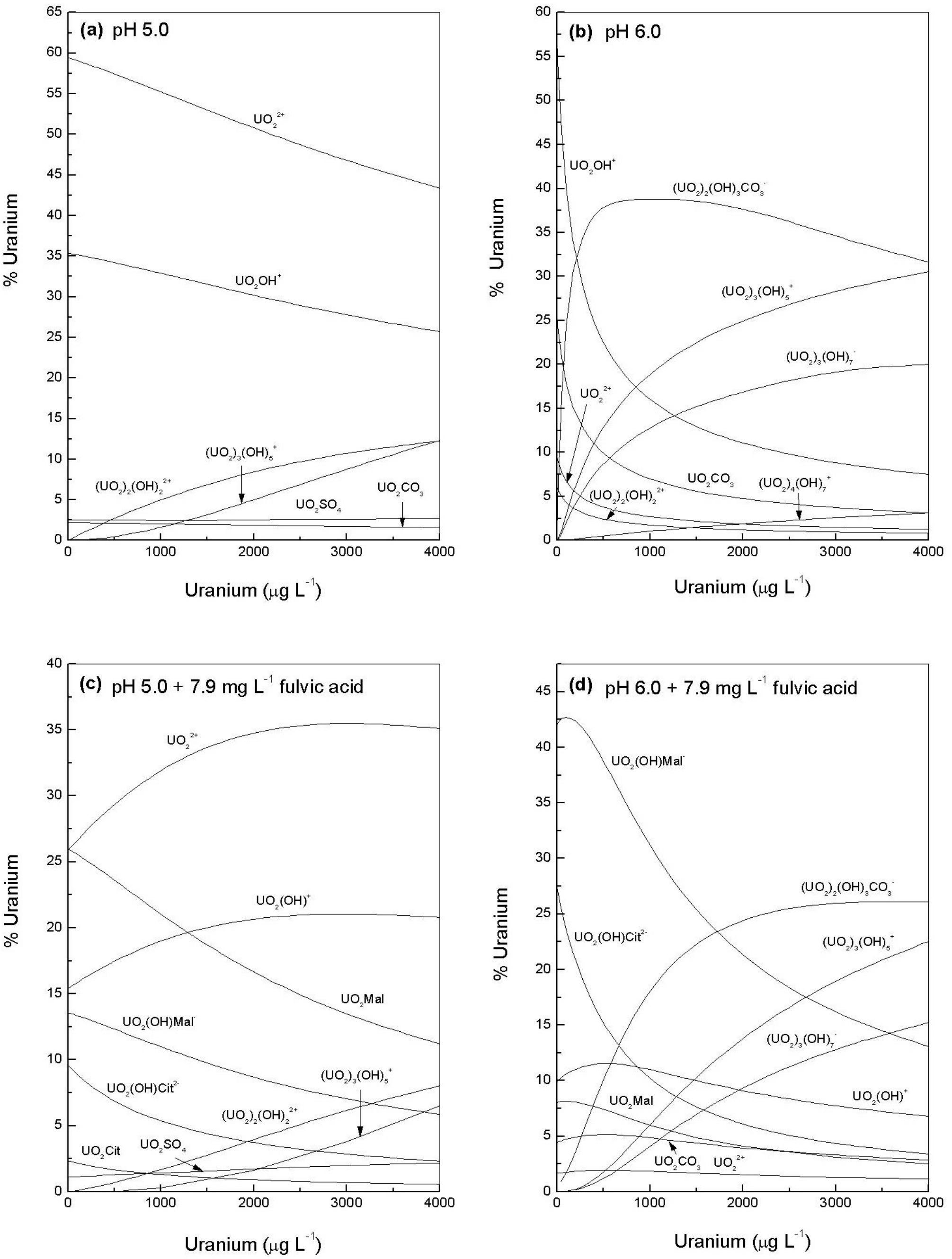

**FIGURE 4.** Predicted speciation (% distribution) of U in synthetic Magela Creek water at (a) pH 5.0 without model fulvic acid (FA); (b) pH 6 without model FA; (c) pH 5 with 7.9 mg $l^{-1}$ model FA; and (d) pH 6 with 7.9 mg $l^{-1}$ model FA.

conditions, the biological response (BR) of *V. angasi* to U was related to the activity of particular U species (i.e., BR $\propto$ 1.86 $\times$ $UO_2^{2+}$ + $UO_2OH^+$) and not the total U concentration (Fig. 5). These results suggest that $UO_2^{2+}$ has nearly a twofold higher binding affinity than $UO_2OH^+$ at the cell membrane surface. No other studies have determined the effects of HS on U uptake by, or toxicity to, aquatic organisms.

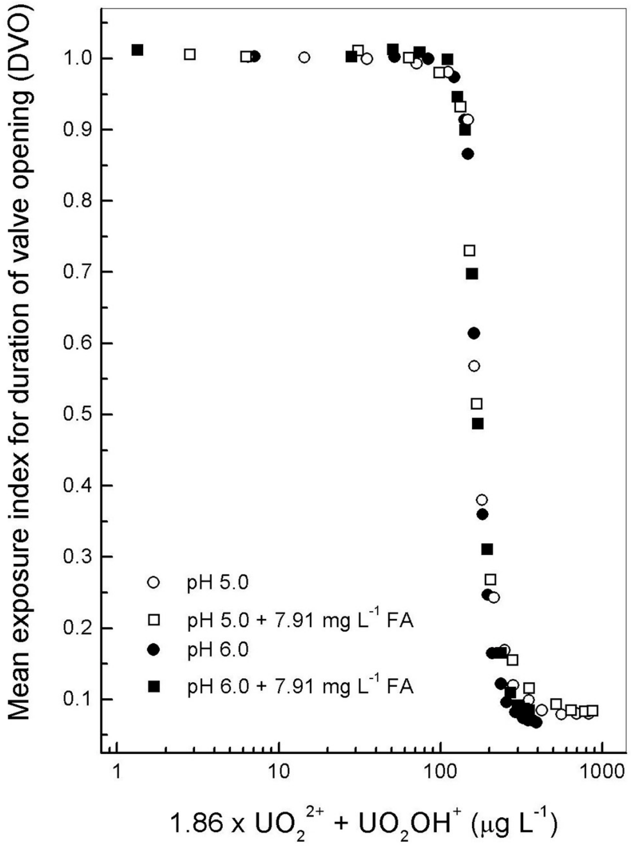

**FIGURE 5.** Concentration-response relationships of the DVO for *V. angasi* expressed in terms of the activities of $UO_2^{2+}$ and $UO_2OH^+$ at pH 5 and 6 with and without model FA (7.9 mg $l^{-1}$). Each plotted point represents the mean response of six bivalves. Error bars and curve fits are excluded for clarity.

Markich et al.[98] also compared the DVO of *V. angasi* to U in natural and synthetic Magela Creek water, matched in terms of major and trace element concentrations, pH (5.5) and natural FA concentration (5.7 mg $l^{-1}$), to test the practical use of the latter in predicting the potential toxicity of U to freshwater organisms in Magela Creek. The results showed that there was no significant ($p > 0.05$) difference in the mean DVO of *V. angasi* exposed to U in both waters (Fig. 6). The above results strongly indicate that the valve movement response of *V. angasi* to U in Magela Creek water can be reliably estimated using customized experimental water. This approach may ultimately improve risk assessment models for the protection of freshwater ecosystems exposed to U. Moreover, the use of a "simple" FA model with customized experimental water requires further verification with other organisms (and metals), including the use of alternative biological endpoints (e.g., survival and uptake) and longer exposures, to determine its true value in predicting the biological effects of U (and other metals) in natural waters. It is perhaps premature to vigorously embrace such a relatively simple approach until these issues have been addressed and any shortcomings exposed.

Ebbs et al.[96] found that the uptake rate of U by the shoots of the pea, *Pisum sativum*, grown hydroponically in low-nutrient solutions, decreased twofold as pH increased from 5 to 6. Speciation modeling of the test solutions showed that this corresponded with a decrease in $UO_2^{2+}$

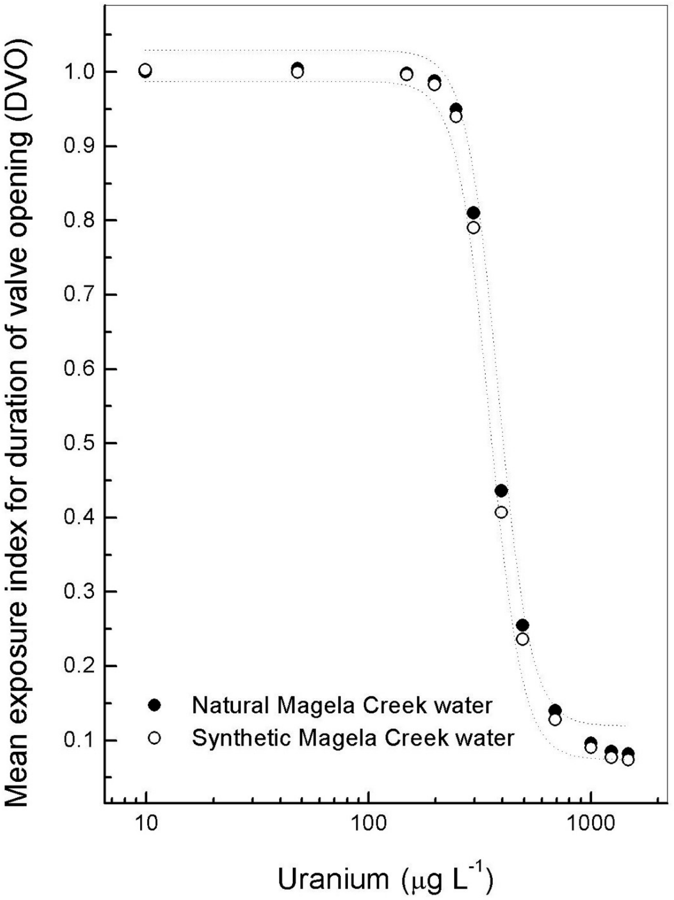

**FIGURE 6.** Concentration-response relationships of the DVO for *V. angasi* exposed to U in natural and synthetic Magela Creek water at pH 5.5 with 5.7 mg $l^{-1}$ fulvic acid. Each plotted point represents the mean response of six bivalves. Error bars are excluded for clarity. The dotted lines represent the upper and lower 95% confidence bands around the sigmoidal curve.

(from 77 to 5%) and an increase in uranyl hydroxide and uranyl carbonate species. The authors postulated that the rate of U uptake by *P. sativum* was governed by the activity of $UO_2^{2+}$ in solution.

The results from Markich et al.[98] and Ebbs et al.[96] apparently differ from the results of Franklin et al.[97], who showed that the toxicity (population growth) of U to the unicellular green alga, *Chlorella* sp., in synthetic Magela Creek water increased twofold (i.e., the 72 h $EC_{50}$ decreased from 78 to 38 μg U $l^{-1}$) when pH increased from 5.7 to 6.5, in the absence of dissolved organic carbon. The speciation of U in this concentration range was predicted to be dominated (70 to 93%) by $UO_2(OH)_3CO_3^-$, with only a very small (<4%) proportion occurring as $UO_2^{2+}$. Given the small proportion of $UO_2^{2+}$ at both pH values, the effect of $H^+$ itself may have been responsible for reducing the toxicity of U to *Chlorella* sp. at pH 5.7. Indeed, Franklin et al.[97] found that intracellular U was twofold lower at pH 5.7 than at 6.5.

Nakajima et al.[102] and Greene et al.[103] showed that, when water hardness (Ca and/or Mg concentration) and alkalinity were held constant, the uptake rate of U by unicellular green algae (*C. regularis* and *C. vulgaris)* was highest at pH 5 to 6, and declined exponentially with increasing pH up to 9, and with decreasing pH down to 3. The increase in pH (i.e., decrease in $H^+$) from 5 to 9 results in a large change in the speciation of U, as predicted using speciation modeling. However, these two effects (i.e., $H^+$ and speciation change) must be uncoupled before the bioavailability of U can be understood. Speciation modeling of the test solutions in the two studies predicted that the relative proportions of $UO_2^{2+}$ and $UO_2OH^+$ declined, while the proportions of uranyl carbonate and polymeric uranyl hydroxide complexes increased, as the pH increased from 5 to 9. Below pH 5 the speciation of U remains constant. Nakajima et al.[102] and Greene et al.[103] postulated that U uptake by *Chlorella* was inhibited at low pH (<5) by protonation of weakly basic binding sites on the algal surface (i.e., $H^+$ competition).

## Effects of Carbonate and Phosphate

Markich et al.[75] found that a fivefold increase in the bicarbonate concentration of synthetic Magela Creek water, at a fixed pH (5) and water hardness (3.5 mg $CaCO_3$ $l^{-1}$), resulted in a 20% reduction in the toxicity (DVO) of U to *V. angasi*. The decrease in U toxicity corresponded to a similar factor of decrease in the calculated percentages of $UO_2^{2+}$ and $UO_2OH^+$, or an increase in $UO_2CO_3$ due to $UO_2^{2+}$ complexation with carbonate. Similarly, Nakajima et al.[102] and Greene et al.[103] showed that the uptake rate of U by unicellular green algae (*C. regularis* and *C. vulgaris)* decreased with increasing carbonate concentration, where both pH and water hardness were held constant. Based on the results of speciation modeling, the authors of both studies postulated that complexation of U by carbonate effectively reduced the activity of $UO_2^{2+}$, and hence, the uptake of U by the algae.

Nakajima et al.[102] and Ebbs et al.[96] showed that the rate of U uptake by unicellular green algae (*C. regularis* and *C. vulgaris*) and the pea (*P. sativum*), respectively, was reduced when phosphate was added to the test solutions. Based on the results of speciation modeling, both authors postulated that complexation of U by phosphate effectively reduced the activity of $UO_2^{2+}$, and hence the rate of U uptake by the test organisms. Fortin et al.[104] used an inverse experimental design in studying the rate of U uptake by the freshwater alga, *Chlamydomonas reinhardtii* at pH 5, to support these results. Based on speciation modeling calculations, the activities of $UO_2^{2+}$, $UO_2OH^+$, and $UO_2(OH)_2$ were kept constant, while the activities of $UO_2HPO_4$ and $UO_2PO_4^-$ were increased. In accordance with the extended free ion activity model[98], the rate of U uptake was unaffected, indicating that the uranyl phosphate complexes were not bioavailable.

## Effect of Water Hardness

Riethmuller et al.[99] found that the toxicity (population growth) of U to green hydra, *Hydra viridissima*, in synthetic Magela Creek water (pH 6 and constant alkalinity, 4 mg $CaCO_3$ $l^{-1}$) decreased twofold (i.e., the 96 h $EC_{50}$ increased from 114 to 219 µg U $l^{-1}$) with a 50-fold increase in water hardness (6.6 to 330 mg $CaCO_3$ $l^{-1}$), added as Ca and Mg sulfate. Similarly, Charles et al.[100] found that a 50-fold increase in water hardness (8 to 400 mg $CaCO_3$ $l^{-1}$) resulted in a fivefold decrease in the toxicity (population growth) of U to the tropical freshwater alga, *Chlorella* sp. (i.e., an increase in the 72 h $EC_{50}$ from 56 µg $l^{-1}$ at 8 mg $CaCO_3$ $l^{-1}$ to 270 µg $l^{-1}$ at 400 mg $CaCO_3$ $l^{-1}$). Speciation modeling calculations in both studies showed that the speciation of U did not significantly ($p > 0.05$) change with increasing water hardness. This suggested that U speciation changes are unlikely to be responsible for the observed decrease in U toxicity with increased water hardness. Thus, the reduction in U toxicity with increased water hardness is most likely due to competition between U and Ca and/or Mg for binding sites on the cell surface of the organisms. In the study by Charles et al.[100], both extracellular and intracellular U concentrations of U were lower at the highest water hardness, indicating that the decreased toxicity of U to *Chlorella* sp. was primarily due to a decrease in U binding at the cell surface, leading to a decreased cellular uptake of U.

Several other studies have determined the effects of increasing water hardness on the toxicity of U to freshwater organisms (fish[105]; water flea[106,107]). However, because these studies confounded the effects of increasing water hardness with increasing alkalinity and pH, the true effects of water hardness (i.e., Ca and/or Mg concentration) on U toxicity could not be discerned. For example, Parkhurst et al.[105] reported that the 96 h $LC_{50}$ for brook trout (*Salvelinus fontinalis*) in soft water (hardness, 35 mg $CaCO_3$ $l^{-1}$; alkalinity, 11 mg $CaCO_3$ $l^{-1}$; pH, 6.7) was 5.5 mg U $l^{-1}$, whereas in hard water (hardness, 208 mg $CaCO_3$ $l^{-1}$; alkalinity, 53 mg $CaCO_3$ $l^{-1}$; pH, 7.5) it was 23.0 mg U $l^{-1}$.

## Implications for Protecting Aquatic Ecosystems

There is reasonable evidence from the literature to indicate that $UO_2^{2+}$ and $UO_2OH^+$ are the major bioavailable forms of U(VI) in water. Uranyl complexes with inorganic ligands (e.g., carbonate or phosphate) and HS apparently reduce the bioavailability of U by reducing the activity of $UO_2^{2+}$ and $UO_2OH^+$. These results have potentially important implications for the protection of aquatic ecosystems. Given the strong binding affinity of U with carbonate, HS, and/or particulate material in surface waters, the fraction of bioavailable U will invariably be small (cf. Figs. 1 and 2). Therefore, the concentrations of total (dissolved and particulate) U in surface waters will usually overestimate the bioavailable U fraction. U guideline values for protecting aquatic ecosystems are also typically based on total U concentrations (e.g., [6]). Therefore, U guidelines values may be overprotective for freshwater ecosystems. The adoption of a tiered speciation scheme[108] that starts simply with a comparison of the guideline value with total metal concentration and increases in complexity via dissolved (filtered) and then bioavailable (speciation modeling or bioassays) metal concentrations[109], will assist in developing U indicators that better reflect biological effects and maintain ecosystem integrity.

## SUMMARY

Although a range of analytical techniques are available for determining the speciation of U in natural surface waters (Table 1), only TRLFS can directly determine specific U species at present. Nevertheless, this technique has limitations, particularly at alkaline pH (8 to 9) and at high ionic strengths (i.e., not useful for estuarine and seawater), and further research is required to extend its application. Electrochemical techniques, such as cathodic stripping voltammetry or chronopotentiometry, provide only an indirect, or operationally defined, measure of U speciation in estuarine or seawater. Due to a lack of analytical techniques to directly determine U species, thermodynamic speciation modeling has been primarily used to provide detailed information on aqueous U species under defined physicochemical conditions. While there are several limitations in using speciation models, the general consensus is that they can provide useful results if applied correctly and with an understanding of the differences between simulated and natural systems. More effort is required to develop new, or improve existing, analytical methodologies to measure U speciation. Only then can modeling calculations of U speciation be objectively evaluated, at least in part.

Due to analytical difficulties, traditional methods for determining the speciation of U in sediments have relied primarily on chemical extraction techniques, in which various phases within the matrix are operationally defined. Although several studies have employed sequential extraction techniques to indirectly determine the speciation of U in sediments or suspended particulate matter, the lack of a standard method has made true comparisons of results difficult. These limitations are minimized by using qualitative or quantitative *in situ* or direct speciation techniques. Some *in situ* spectroscopic techniques, such as X-ray absorption spectroscopy, can provide qualitative information that is selective for one oxidation state (U[IV] or U[VI]) or mineral phase of U in sediments. In practice, a combination of these techniques has been used to probe the speciation of U in sediments.

There is little information relating the speciation of U to its bioavailability in aquatic systems. All available information has been derived using simple, chemically defined experimental freshwaters, rather than natural waters. No data are available for estuarine or seawater. Furthermore, there are no available data on the relationship between U speciation and bioavailability in sediments. These two gaps clearly require further work. Based on the results of freshwater studies, there is reasonable evidence to indicate that $UO_2^{2+}$ and $UO_2OH^+$ are the major bioavailable forms of U(VI). Uranyl complexes with inorganic ligands (e.g., carbonate or phosphate) and HS apparently reduce the bioavailability of U by reducing the activity of $UO_2^{2+}$ and $UO_2OH^+$. The majority of studies have used the results from speciation modeling to support these conclusions.

## ACKNOWLEDGMENTS

Paul Brown, Tim Payne, Ross Jeffree, Gillian Peck, John Harries, Jenny Stauber, Simon Apte, Graeme Batley, Stuart Simpson and two anonymous referees provided constructive comments on previous manuscripts.

## BIOSKETCH

**Dr. Scott Markich** is a Research Scientist in the Environment Division of the Australian Nuclear Science and Technology Organisation (ANSTO). He holds a B.Sc. from Sydney University and a Ph.D. from the University of Technology in Sydney. His research work has focussed primarily on the ecological impacts of contaminants, with an emphasis on mine waste waters (e.g., Ranger Uranium mine, Rum Jungle and Brukunga) in freshwater systems. This work has focussed mainly on bioaccumulation and toxicity studies of metals and radionuclides with freshwater organisms. The biology has been integrated with detailed studies of trace metal speciation in freshwaters. Dr. Markich is currently coordinating research into understanding the mechanisms of metal interaction with aquatic organisms. These data have been used in the derivation of practical water quality guidelines for the protection of aquatic ecosystems in Australia and New Zealand. He has over 40 research publications in the field.
